# Ileus tube-assisted endoscopic detorsion as a useful initial management for cecal volvulus in a patient with septic shock

**DOI:** 10.1055/a-2291-9675

**Published:** 2024-04-09

**Authors:** Hideaki Kazumori, Takashi Sato

**Affiliations:** 1 13838 Department of Gastroenterology, Matsue Seikyo General Hospital, Matsue, Japan; 2 13838 Department of Surgery, Matsue Seikyo General Hospital, Matsue, Japan

A 70-year-old man with abdominal pain and vomiting came to our emergency department, and was found to be in a serious condition. Arterial blood gas analysis showed a pH of 7.24, with bicarbonate 12.9 mmol/L, lactate 6.51 mmol/L, and anion gap 26.7 mmol/L, and therefore lactic acidosis was considered. Laboratory results found elevated aspartate aminotransferase at 463 U/L, alanine aminotransferase at 371 U/L, creatine kinase at 1566 U/L, and lactate dehydrogenase at 779 U/L. Computed tomography findings indicated that his cecum was grossly dilated and inverted, and mesenteric whirl signs were also noted ( Fig. 1 ). A diagnosis of cecal volvulus with intestinal necrosis was made.

**Fig. 1:**
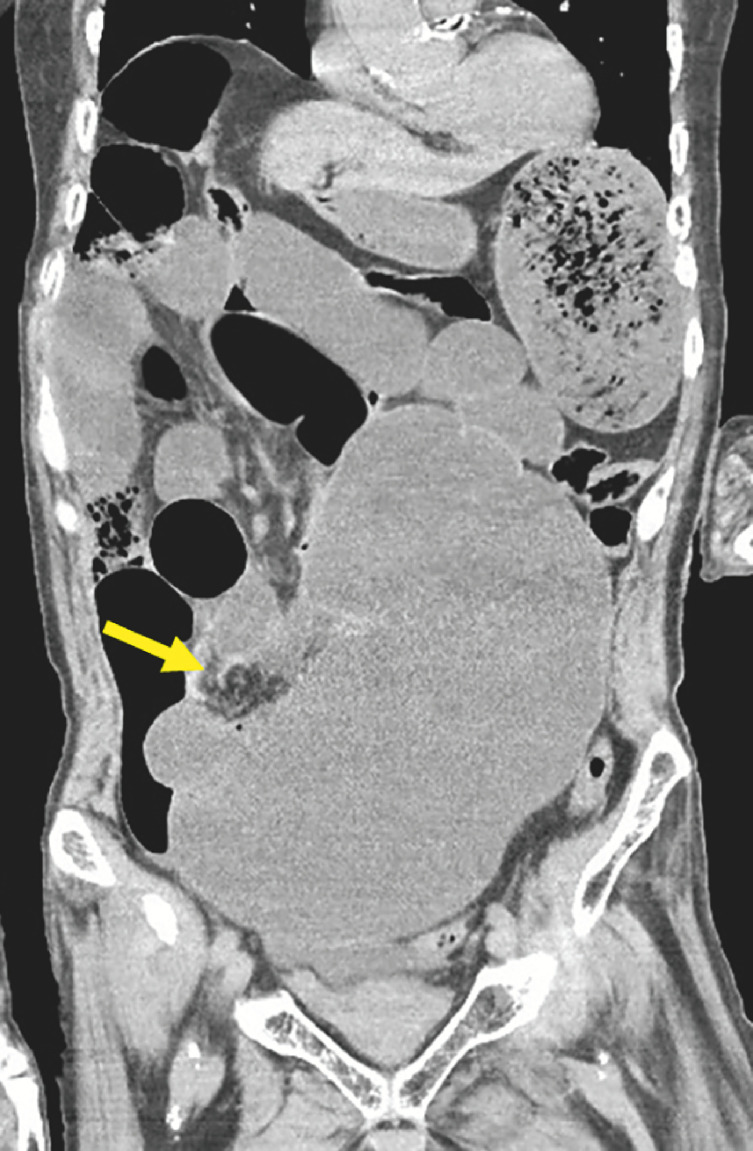
Computed tomography scan showing a grossly dilated and inverted cecum, along with mesenteric whirl signs (arrow).

Despite adequate fluid infusion and noradrenaline administration at high dosage, the patient’s blood pressure gradually dropped to 60/40 mmHg and he lost consciousness. An emergency laparotomy was planned, but his general condition was not considered adequate to undergo this, so endoscopic detorsion of the cecal volvulus was therefore attempted. A colonoscopy was performed without the patient being sedated to prevent a further decrease in his blood pressure from such medication, and because he was already unconscious. When the colonoscope (PCF-H290ZI, with attachment D-201-13404; Olympus Corp., Tokyo, Japan) was inserted into the ascending colon, white and dark purple coloring of the twisted site was seen, along with obstruction that prevented advancement of the colonoscope ( Fig. 2 and Fig. 3 ). After insertion of a guidewire, a thin ileus tube (Hydrophilic Long Intestinal Tube Type CP-II, 16 Fr; Create Medic Co., Ltd., Kawasaki, Japan) was inserted into the cecum. The colonoscope was then successfully advanced into the cecum using the tube as a guide ( Video 1 ). The torsion was reduced by suctioning gas and twisting the cecum, and the patient’s clinical status gradually improved. Thereafter, a laparotomy was performed as previously planned and the patient was discharged without complications.

**Fig. 2:**
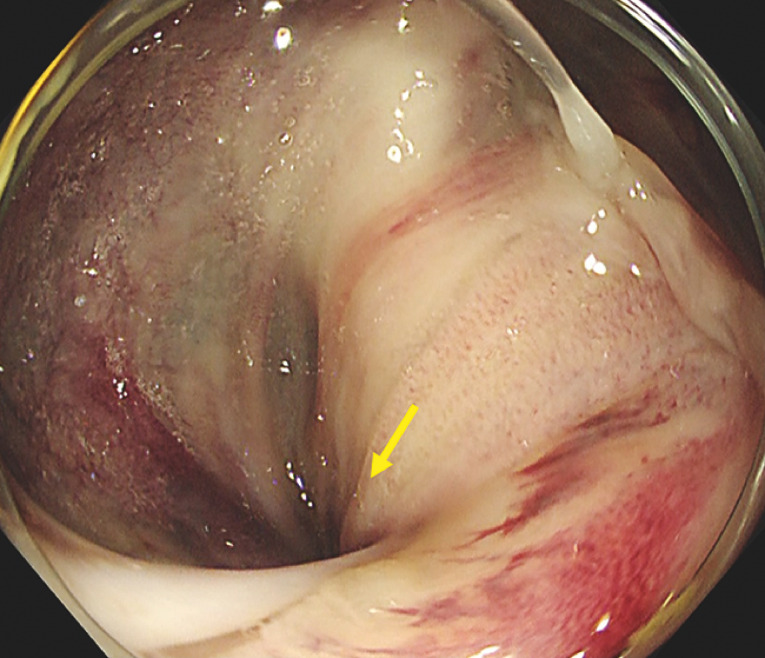
Colonoscopic image of the ascending colon, showing the twisted area (arrow), along with white and purple discoloration of the mucosa, similarly showing a twisted pattern.

**Fig. 3:**
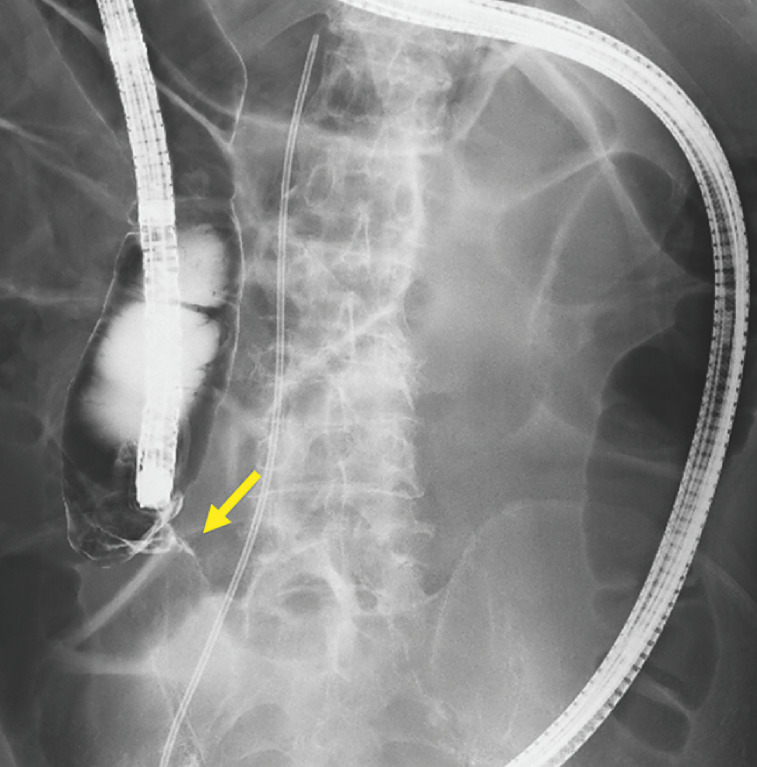
Fluoroscopic image of the ascending colon with amidotrizoate acid used as a contrast agent showing twisting and obstruction of the ascending colon (arrow), with contrast medium unable to pass through the twisted site.

Colonoscopy is performed in a patient with a cecal volvulus showing white and dark purple coloring, and obstruction in the twisted portion of the ascending colon, which is successfully treated by advancing a colonoscope into the cecum using an ileus tube as a guide.Video 1

Unlike for a sigmoid volvulus, endoscopic detorsion is not generally recommended as initial management for a cecal volvulus, because of its low success rates and the high risk of intestinal perforation 1 . In the present patient, insertion of an ileus tube loosened the torsion and provided a guide for advancement of the colonoscope. When emergency surgery is difficult, initial management by ileus tube-assisted endoscopic detorsion should be considered as a potentially lifesaving procedure.

Endoscopy_UCTN_Code_TTT_1AQ_2AF

## Notes

Endoscopy E-Videos https://eref.thieme.de/e-videos
*E-Videos* is an open access online section of the journal *Endoscopy* , reporting on interesting cases and new techniques in gastroenterological endoscopy. All papers include a high-quality video and are published with a Creative Commons CC-BY license. Endoscopy E-Videos qualify for HINARI discounts and waivers and eligibility is automatically checked during the submission process. We grant 100% waivers to articles whose corresponding authors are based in Group A countries and 50% waivers to those who are based in Group B countries as classified by Research4Life (see: https://www.research4life.org/access/eligibility/ ). This section has its own submission website at https://mc.manuscriptcentral.com/e-videos .

## References

[1] Kapadia MR Volvulus of the small bowel and colon Clin Colon Rectal Surg 2017 30 40 45 10.1055/s-0036-1593428 28144211 PMC5179272

